# The Identification of a Tumor Infiltration CD8+ T-Cell Gene Signature That Can Potentially Improve the Prognosis and Prediction of Immunization Responses in Papillary Renal Cell Carcinoma

**DOI:** 10.3389/fonc.2021.757641

**Published:** 2021-11-10

**Authors:** Jie Wang, Meiying Huang, Peng Huang, Jingjie Zhao, Junhua Tan, Feifan Huang, Ruiying Ma, Yu Xiao, Gao Deng, Liuzhi Wei, Qiuju Wei, Zechen Wang, Siyuan He, Jiajia Shen, Suren Sooranna, Lingzhang Meng, Jian Song

**Affiliations:** ^1^ Center for Systemic Inflammation Research (CSIR), School of Preclinical Medicine, Youjiang Medical University for Nationalities, Baise, China; ^2^ Department of Renal Diseases, Affiliated Hospital of Youjiang Medical University for Nationalities, Baise, China; ^3^ Life Science and Clinical Research Center, Affiliated Hospital of Youjiang Medical University for Nationalities, Baise, China; ^4^ School of Pharmacy, Youjiang Medical University for Nationalities, Baise, China; ^5^ Department of Metabolism, Digestion and Reproduction, Imperial College London, Chelsea & Westminster Hospital, London, United Kingdom; ^6^ Department of Radiation Oncology, Renji Hospital, School of Medicine, Shanghai Jiao Tong University, Shanghai, China

**Keywords:** tumor infiltration immune cells, prognostic model, papillary renal cell carcinoma, CD8+ T cell, scRNA-seq

## Abstract

**Background:**

CD8+ T cells, vital effectors pertaining to adaptive immunity, display close relationships to the immunization responses to kill tumor cells. Understanding the effect exerted by tumor infiltration CD8+ T cells in papillary renal cell carcinoma (papRCC) is critical for assessing the prognosis process and responses to immunization therapy in cases with this disease.

**Materials and Approaches:**

The single-cell transcriptome data of papRCC were used for screening CD8+ T-cell-correlated differentially expressed genes to achieve the following investigations. On that basis, a prognosis gene signature associated with tumor infiltration CD8+ T cell was built and verified with The Cancer Genome Atlas data set. Risk scores were determined for papRCC cases and categorized as high- or low-risk groups. The prognosis significance for risk scores was assessed with multiple-variate Cox investigation and Kaplan–Meier survival curves. In addition, the possible capability exhibited by the genetic profiles of cases to assess the response to immunization therapy was further explored.

**Results:**

Six hundred twenty-one cell death-inhibiting RNA genes were screened using single-cell RNA sequencing. A gene signature consisting of seven genes (*LYAR*, *YBX1*, *PNRC1*, *TCF25*, *MYL12B*, *MINOS1*, and *LINC01420*) was then identified, and this collective was considered to be an independent prognosis indicator that could strongly assess overall survival in papRCC. In addition, the data allowed papRCC cases to fall to cohorts at high and low risks, exhibiting a wide range of clinically related features as well as different CD8+ T-cell immunization infiltration and immunization therapy responses.

**Conclusions:**

Our work provides a possible explanation for the limited response of current immunization checkpoint-inhibiting elements for combating papRCC. Furthermore, the researchers built a novel genetic signature that was able to assess the prognosis and immunotherapeutic response of cases. This may also be considered as a promising therapeutic target for the disease.

## Introduction

Renal cell carcinoma (hereinafter abbreviated as RCC) is one of the commonest malignancies in kidney cancer, with clear cell renal carcinoma (ccRCC) accounting for more than 70% of RCC cases. Papillary RCC (papRCC) is the second most common histologic subtype after ccRCC, accounting for approximately 15% of RCC (1). For ccRCC, immunization therapy was approved for use in cases with metastatic tumors by virtue of several successful systematic studies and randomized controlled trials (2). However, little has been done with respect to identifying and validating the risk profiles associated with papRCC prognosis. This has been a key obstacle to the development of more stable and reliable prognosis markers for papRCC and, thus, to the development of appropriate therapeutic strategies to combat this disease.

For aggressive cancers, tumor infiltration of T cells refers to the most prioritized immunization cells for an effective cancer targeting. T-cell infiltration was shown to be an excellent prognosis biological marker in terms of survival of cases with ovarian cancer, colorectal cancer, and glioblastoma (3). However, a substantial level of CD8+ T cells in papRCC often indicates a poorer prognosis compared with many other forms of cancer. Previous studies have demonstrated that tumor infiltration CD8+ T cells are the primary immunization cells in papRCC as well as indicators of poor prognosis (4, 5). These opposing effects suggest that there may be various CD8+ T-cell subpopulations or some tumor infiltration CD8+ T cell dysfunction in the papRCC immunization environment. Thus, immunogene-correlated tumor infiltration CD8+ T cell may act as a vital target for identifying genetic signatures that may improve immunotherapeutic responses.

A single-cell RNA sequencing data set was explored in this study for a comprehensive assessment of the different subpopulations of immunization cell and finding a CD8+ T-cell-type particular gene in papRCC. Using a combination of RNA-seq data from a large number of papRCC cases and their corresponding clinically related data, the researchers built a gene signature for tumor infiltration CD8+ T cells with multiple machine learning algorithms. Furthermore, the researchers validated this risk-correlated gene signature using the gene expression profiles and clinically related data from an additional, independent Gene Expression Omnibus (GEO) data set. The genetic signature obtained may provide future targets for increasing our knowledge of papRCC. It may also enhance the efficacy of immunization checkpoint blockade therapies for the disease.

## Material and Approaches

### Human Biopsies

Cancerous biopsies were isolated from patients, to validate the feature genes identified by sequencing. In total, 8 samples were isolated from ccRCC patients and 10 were isolated from papRCC patients after surgery.

### CD8+ T-Cell Estimation Within ccRCC and papRCC

For exploring the relationship between tumor infiltration CD8+ T cell and clinically related prognosis of renal carcinoma, the researchers utilized the Tumor Immunization Estimation Resource (TIMER2.0) database (http://timer.comp-genomics.org/). This was employed to analyze immunization infiltration in different cancer types by multiple immunization deconvolution approaches, which provides Cox regression investigation data and Kaplan–Meier survival curves for estimating the prognosis significance of the relevant immunization infiltrate within a wide range of cancer (6).

### Identification of CD8+ T-Cell-Correlated Immunization-Correlated Gene Within Cancerous Human Kidneys

There were 72,501 single kidney cell transcriptomes including ccRCC (*n* = 3) and papRCC (*n* = 1) and adult kidneys (*n* = 5) (7) that were used. ccRCC and papRCC groups consisted of paraneoplastic and tumor samples.

### Single-Cell RNA-Seq Information Investigation

Single-cell data from ccRCC paraneoplastic, ccRCC tumor, papRCC paraneoplastic, papRCC tumor, and non-tumor samples were integrated and normalized using the SCTransform approach and then analyzed by conducting PCA (https://satijalab.org/seurat/v3.1/integration.html) (8). This was also conducted for the integrated data sets, and cluster investigation was carried out using UMAP. The cluster investigation of single-cell data was carried out with Seurat’s graph-based clustering approach, and the resolution of the FindClusters feature was set to 0.1. Subsequently, the clusters were visualized using Uniform Manifold Approximation and Projection (UMAP), version 0.2.6.0 graphics. The R software package, Seurat (version 2.3.4), was used for the data investigation. For quality control purposes, unique molecular identifier (UMI) counts of less than 500 and those with double multiples were removed. Furthermore, cells with percentages of mitochondrial and ribosomal genes of less than 5% and 50%, respectively, were filtered out.

### ccRCC and papRCC Large-Scale RNA-Seq Data Set Collection and Processing

The majority of RNA sequencing data sets for ccRCC and papRCC cases as well as their corresponding clinically related data originated from the TCGA database (https://portal.gdc.cancer.gov/) and consisted of 286 papRCC samples. TCGA samples were randomly separated into the training group and the testing group. Subtype information of papRCC samples was obtained from the cBioPortal website. The raw gene expression data set was processed. Probe ID had the annotation to gene employing the software package of Bioconductor, and the relevant platform annotation profile and the raw matrix data received log2 transformation and quantile normalization. Clinically related samples with missing values were excluded from the final investigation.

### Building the CD8+ T-Cell-Correlated Gene Signature

Single-cell data were classified into cell types and divided according to their respective tissue sources. The corresponding transcriptome investigation data were compared to screen differentially expressed gene (DEGs) and to increase the efficiency of the study. A min.pct >0.25 and |Log2 (FC)| >0.5 were used for the subsequent investigation.

The correlation between papRCC tumor infiltration CD8+ T-cell-correlated DEGs and overall survival time in TCGA papRCC cases was analyzed. Univariate Cox regression investigation was carried out to identify genes associated with survival (*p*-value < 0.05). Subsequently, the significance of candidate genes was selected using VIMP in a randomized survival forest (RSF) algorithm. A risk score model with DEGs taken was built using multiple-variate Cox regression approaches. In addition, the Kaplan–Meier test was employed for a number of gene features and *p*-values (log) were determined. Receiver operating characteristic (ROC) investigation was carried out for 3- and 5-year overall survival rates, and AUC was determined to assess the specificity and sensitivity of the gene signature. In addition, to examine the robustness of the results, the papRCC tumor infiltration CD8+ T-cell-correlated gene signature was further validated.

### Flow Cytometry Analysis

Briefly, biopsies were cut into pieces and digested with collagenase IV (20 mg/ml, Gibco, #17104-019) at 37 C for 1 h and incubated for another 10 min after adding 2 mM EDTA. The digested biopsies were filtered through a 100-μm cell strainer to obtain a single-cell suspension.

The cells were incubated with Fcγ receptor blocker (Invitrogen, #14-9161-73) for 10 min to block unspecific binding. Then, fluorescent-conjugated antibodies were added for surface staining. A commercial Cytofix/Cytoperm kit (BD, #554717) was used according to the instructions of the manufacturer for intracellular staining.

Fluorescent-conjugated antibodies used in this study were as follows: PE mouse-anti-human CD8 (Invitrogen, #MA1-12030), APC mouse-anti-human LYAR (LSBio, #LS-C719928), FITC rabbit-anti-human PNRC1 (LSBio, #LS−C455780), APC rabbit-anti-human RNF115 (Bioss, #bs-6757R-APC), Cy5 rabbit-anti-human YBX1 (Bioss, #bs-5921R-Cy5), FITC rabbit-anti-human TCF25 (Bioss, #bs-9604R-FITC), Cy7 rabbit-anti-human MYL12B (Bioss, #bs-19147R-Cy7), and A488 rabbit-anti-human MINOS1 (Bioss, bs-15029R-A488).

### Group Investigation

To assess the relationship of risk group as well as clinically related characteristics, group investigation was carried out in terms of papRCC clinically related variable with a large variety, covering ages, genders, stages, histological types, and survival. In addition, to assess the prognosis value, multiple-variate Cox regression investigation was carried out to determine if risk scores were of prognosis significance not determined by other clinically related variables.

### Statistical Investigation

With R software (version 3.6.0), this study exploited statistically related investigations. Kaplan–Meier tests and ROC analyses were carried out with the “survivor” and “survROC” software packages (9). Optimal cutoff values were determined using the “survminer” package (10). The researchers employed univariate and multiple-variate Cox regression investigations to assess the prognosis factors of interest. Hazard ratios (HR) and 95% confidence intervals (95% CI) were determined for the prognosis factors. In all statistical tests, *p <*0.05 was considered statistically significant.

## Results

### Opposite Results of CD8+ T Cells in papRCC in Comparison With ccRCC

**Figure 1:**
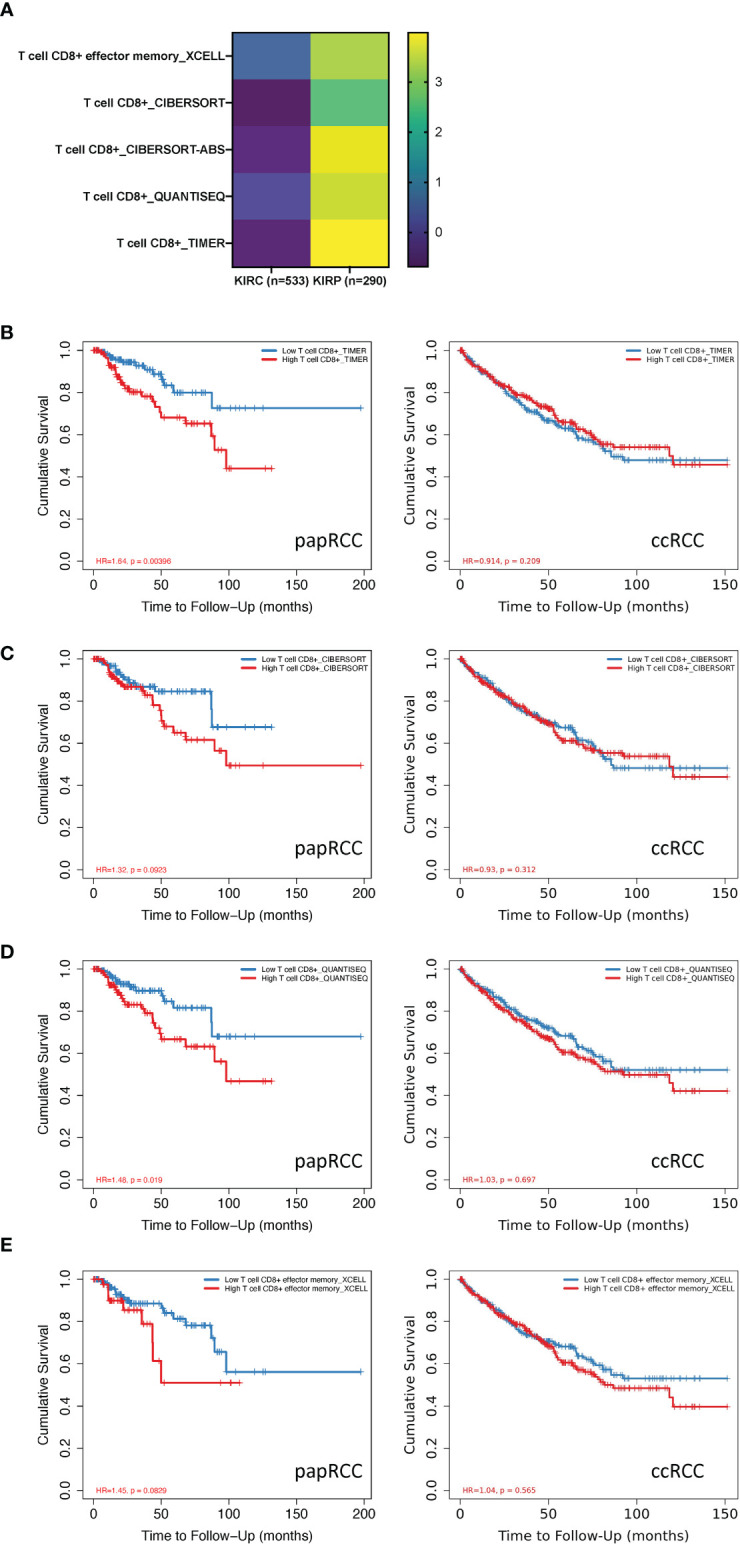
Prognosis value of CD8+ T cells in papillary renal cell cancer (papRCC). **(A)** Heat map of multiple-variate Cox proportional risk model in terms of CD8+ T cell within papRCC. *Z*-score represents the risk score. **(B)** Kaplan–Meier survival curve investigation of CD8+ T cells in papRCC and ccRCC with TIMER **(B)**, CIBERSORT **(C)**, CIBERSORT-ABS **(D)**, XCELL **(E)**, and QUANTISEQ approaches.

Multiple immunization deconvolution approaches including “XCELL” (11), “TIMER” (6), “QUANTISEQ,” (12) “CIBERSORT-ABS,” and “CIBERSORT” (13) were used for estimating immunization infiltration in papRCC and ccRCC. With the univariate Cox proportional risk model, we unexpectedly discovered that a substantial amount of CD8+ T-cell infiltration was beneficial for cases with ccRCC, whereas increased CD8+ T-cell tumor infiltration posed a risk for papRCC cases (**Figure 1A**). Kaplan–Meier curves also showed that the survival times of the high tumor infiltration CD8+ T-cell group were significantly shorter than those for the low tumor infiltration CD8+ T-cell group with respect to papRCC, regardless of the deconvolution approach used (**Figures 1B–F**).

### scRNA-Based DEG Identification

**Figure 2:**
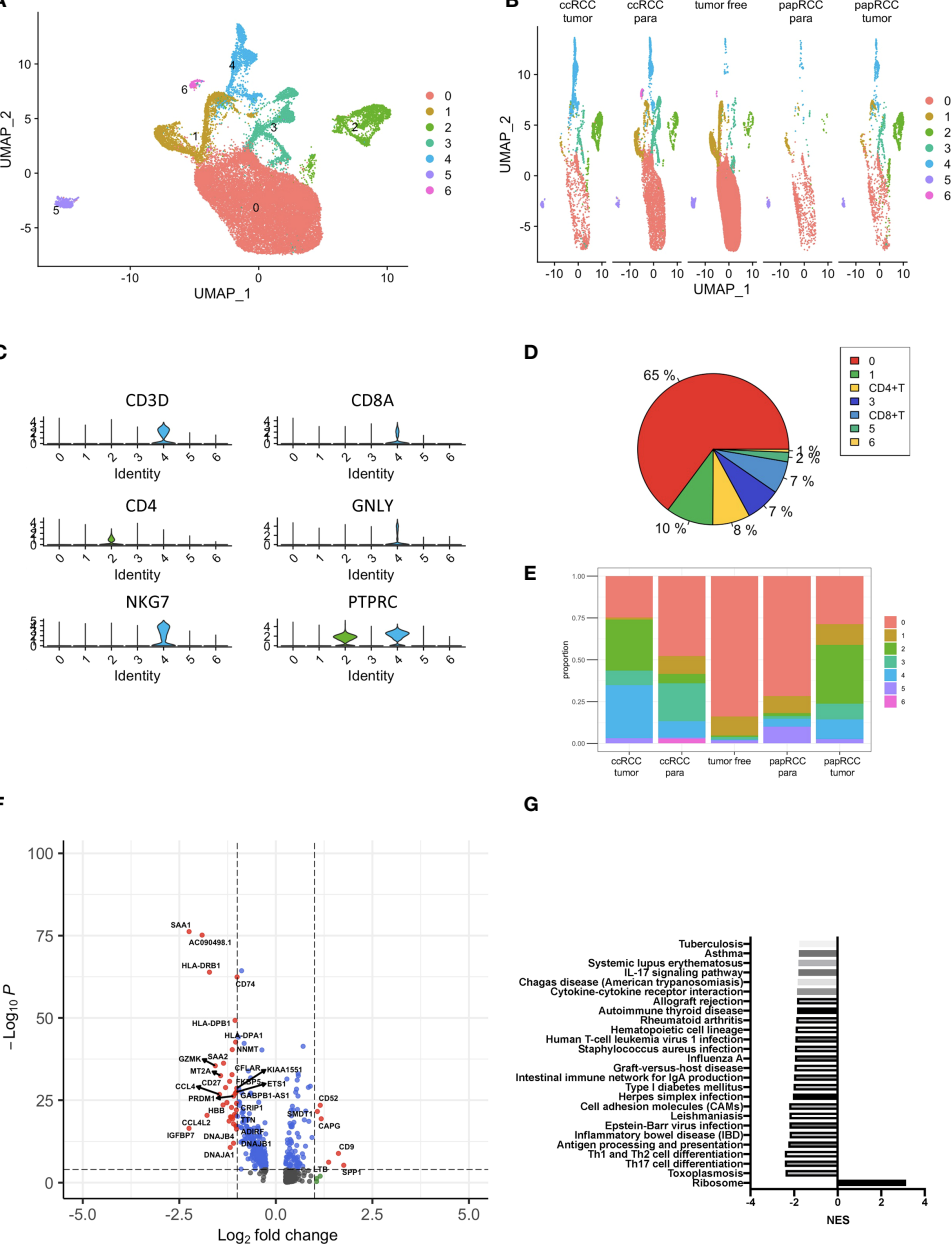
Identification of papRCC tumor infiltration CD8+ T-cell-correlated genes. **(A)** View of a single-cell sample from a RCC case; annotated UMAP plots to identify a total of eight different cell types including epithelial cells, malignant cells, monocytes/macrophages, dendritic cells, CD8+ T, malignant cells, unknown cells, and endothelial cells. **(B)** Views of single cell from tumor-free, ccRCC paraneoplastic, ccRCC tumor, papRCC paraneoplastic, and papRCC tumor samples, respectively. **(C)** Violin plots to demonstrate CD8+ T cells. **(D)** Pie charts of the seven different cell types. **(E)** Bar graphs of the cell proportions of eight different cell types from tumor-free, ccRCC paraneoplastic, ccRCC tumor, papRCC paraneoplastic, and papRCC tumor samples, respectively. **(F)** Volcano plot of the differentially expressed genes (DEGs) in papRCC tumor infiltration CD8+ T cells. **(G)** Bar graph showed the results of KEGG pathway enrichment of DEGs in the papRCC tumor infiltration CD8+ T cells.

scRNA data consisted of 72,501 tumor and non-tumor cells from papRCC, ccRCC, and non-tumor samples. With the UMAP algorithm, these mixtures could be unambiguously classified into eight cell clusters, including epithelial, malignant, dendritic, CD8+ T, malignant, unknown, and endothelial cells as well as monocytes/macrophages (**Figures 2A, B**). The single-cell clusters could also distinguish as to whether they were from ccRCC paraneoplastic or tumor and papRCC paraneoplastic, tumor or non-tumor samples, respectively (**Figure 2C**). The pie chart shows that CD8+ T cells are an important fraction of the renal immunization environment (**Figure 2D**), and the bars indicate that CD8+ T cells account for the major immunization cell infiltration into papRCC as well as ccRCC tumors (**Figure 2E**). Subsequently, 621 papRCC tumor infiltration CD8+ T-cell-correlated DEGs were screened based on the selection criteria set out in the *Method and Approaches* section (**Figure 2F**). The results of KEGG enrichment of these DEGs indicated that papRCC downregulated many of the T-cell function significantly, indicating a complex defective profile of the tumor-infiltrated T cells in the papRCC (**Figure 2G**).

### Gene Signature From the Infiltrated CD8+ T Cells

**Figure 3:**
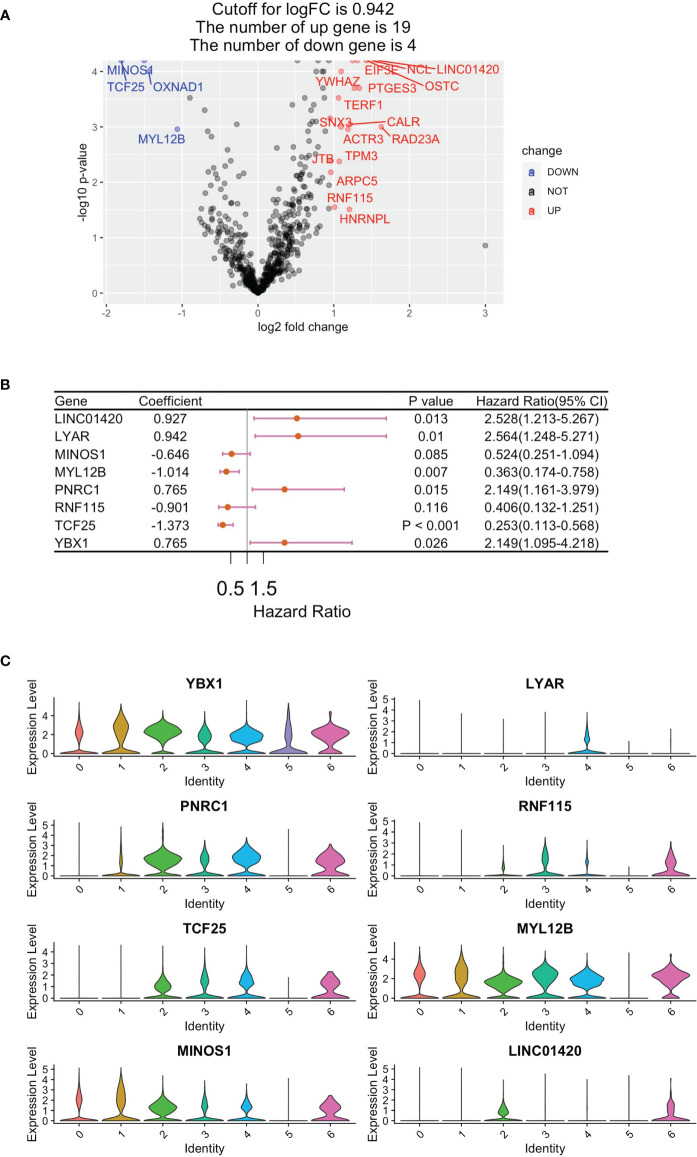
Construction of a CD8+ T-cell-correlated prognosis gene signature. **(A)** Volcano plot showing Cox regression investigation of survival-correlated papRCC-infiltrating CD8+ T-cell DEGs. **(B)** Forest plot lines of the top genes as screened by random survival forest investigation. **(C)** Violin plots showing the expression of the top genes in different cell types.

In total, RNA sequencing data and clinically related data from 283 eligible papRCC cases were obtained from the TCGA data sets. A total of 621 DEGs were filtrated for univariate Cox regression study and 23 of these correlated with papRCC survival significantly (*p* < 0.05) (**Figure 3A**). With the RSF algorithm, the top significant genes, *LYAR*, *YBX1*, *PNRC1*, *RNF115*, *TCF25*, *MYL12B*, *MINOS1* and *LINC01420*, were screened (**Figure 3B**). Violin plots of the scRNA data set revealed high expression levels of these genes (**Figure 3C**).

**Figure 4:**
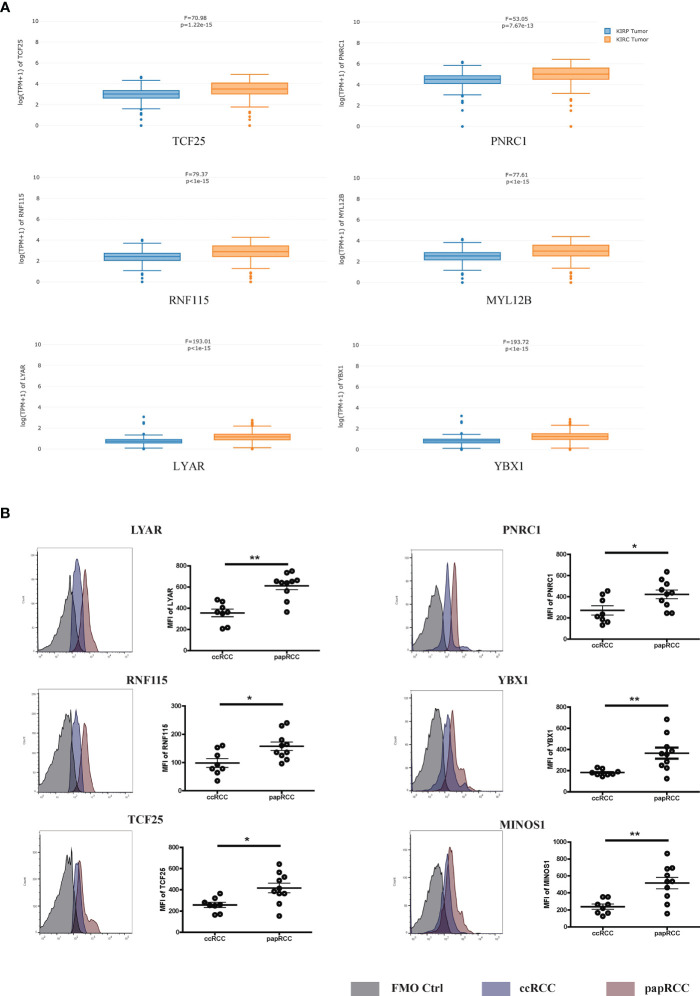
Expression levels of prognosis signature markers in the ccRCC- and papRCC-infiltrated CD8+ T cells. **(A)** Boxplots compare the gene expression of prognosis signature markers in the ccRCC- and papRCC-infiltrated CD8+ T cells. **(B)** Histograms compare the protein levels of prognosis signature markers in the ccRCC- and papRCC-infiltrated CD8+ T cells. *p<0.05 and **p<0.01.

To investigate the gene expression of the signature genes in the samples from a large amount of patient samples, we performed the cell-type-level expression analysis using GEPIA (Gene Expression Profiling Interactive Analysis) (http://gepia2021.cancer-pku.cn/sub-expression.html). We found most of the signature genes in the analysis, which displayed a higher level in papRCC-infiltrating CD8+ T cells compared with ccRCC-infiltrating CD8+ T cells (**Figure 4A**). Only MINOS1 and LINC01420 were not detectable. However, we could validate that the level of MINOS1 on CD8+ T cells isolated from papRCC biopsy samples is higher than the cells from ccRCC samples. Also, we detected a higher level of LYAR, YBX1, PNRC1, RNF115, and TCF25 on papRCC CD8+ T cells compared with that on ccRCC CD8+ T cells (**Figure 4B**), while MYL12B and LINC01420 antibodies were not available. These data suggested a specificity of the current signature genes for papRCC CD8+ T cells.

**Figure 5:**
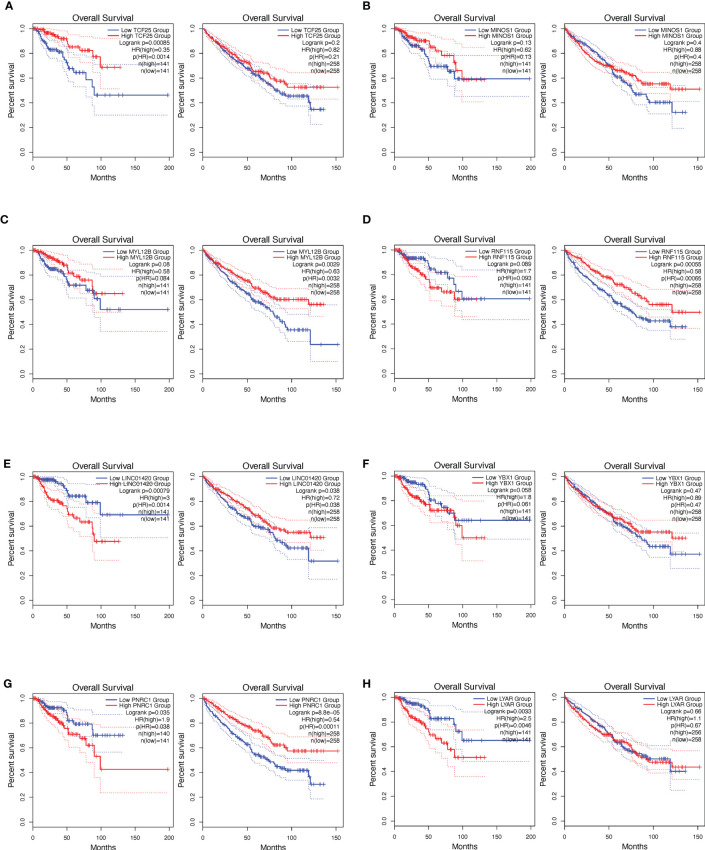
Survival analysis based on the single signature gene. Kaplan–Meier curves show the risk definition of TCF25 **(A)**, MINOS1 **(B)**, MYL12B **(C)**, RNF115 **(D)**, LINC01420 **(E)**, YBX1 **(F)**, PNRC1 **(G)**, and LYAR **(H)** in the papRCC (left column) and ccRCC (right column) TCGA samples.

To study the clinical relevance of each individual gene of the signature, we performed survival analysis based on the expression status of the gene in the TCGA data set of papRCC (282 samples) or ccRCC (516 samples) and plotted a Kaplan–Meier curve (**Figure 5**). Consistent with previous hazard ratio analyses, TCF25, MYL12B, and MINOS1 expressions correlated with a reduced risk (**Figures 5A–C**), while LYAR, YBX1, PNRC1, and LINC01420 showed a high risk (**Figures 5E–H**). The only exceptional one was RNF115, which showed a contradicting result in the multi-gene and single-gene analyses (**Figure 5D**). Concerning the less significance of RNF115 in the hazard ratio analyses, we excluded RNF115 from the gene signature.

**Figure 6:**
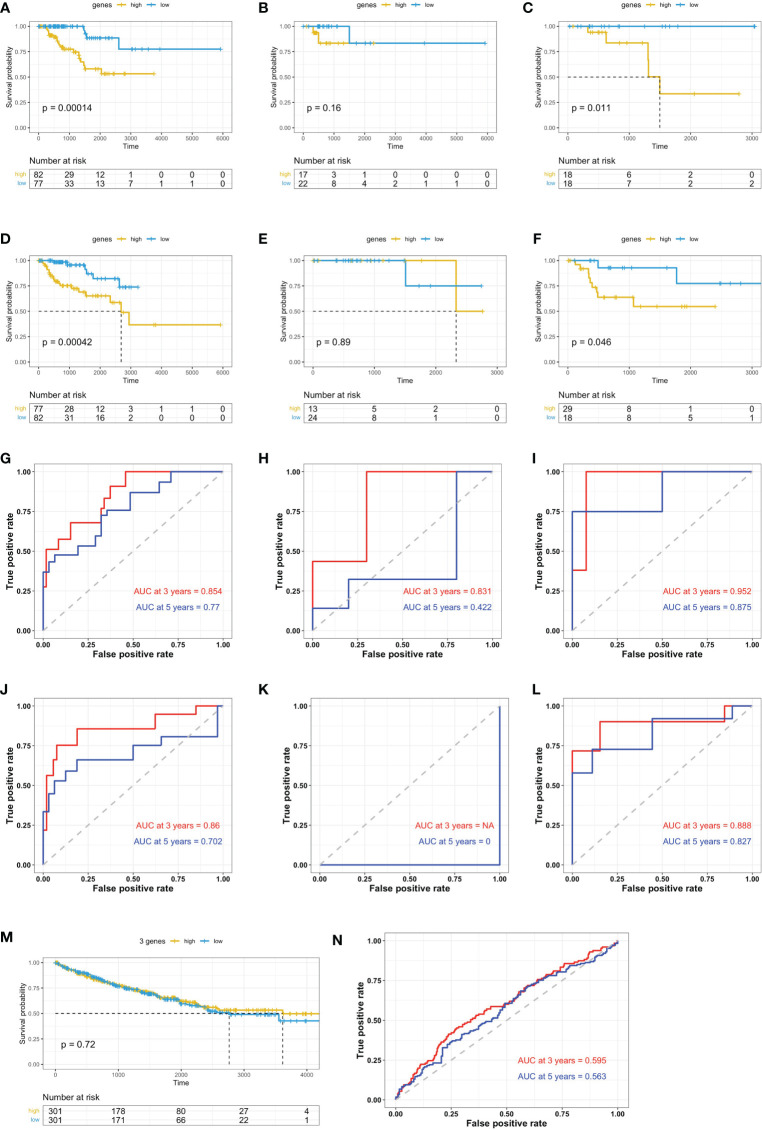
Validation of prognosis gene labels for papRCC and subtypes. Kaplan–Meier (KM) investigation of the risk group defined with CD8+ T-cell-correlated gene tags in the TCGA training data set for **(A)** the general papRCC, **(B)** type 1 papRCC, and **(C)** type 2 papRCC. KM investigation of the risk model for CD8+ T-cell-correlated gene labels in the TCGA testing data set for **(D)** the general papRCC, **(E)** type 1 papRCC, and **(F)** type 2 papRCC. Three- and 5-year receiver operating characteristic curves from the TCGA training data set for **(G)** the general papRCC, **(H)** type 1 papRCC, and **(I)** type 2 papRCC. Three- and 5-year ROC curves from the TCGA testing data set for **(J)** the general papRCC, **(K)** type 1 papRCC, and **(L)** type 2 papRCC. **(M)** KM investigation of the risk model for CD8+ T-cell-correlated gene labels in the TCGA data set of ccRCC. **(N)** Three- and 5-year ROC curves from the ccRCC TCGA data set.

A risk scoring system was then built using these seven genes by applying multiple-variate Cox investigation on the TCGA data set. Based on the formula obtained, a risk score could be determined for each case. The papRCC cases in the TCGA data set were then divided into high-risk and low-risk groups by applying the optimal cutoff values for the risk scores. Kaplan–Meier curves showed that cases in the high-risk group had shorter survival times than those in the low-risk group (**Figure 6A**). Using subtype information from cBioPortal website, we could divide the samples into type 1 and type 2 papRCC. The current risk markers worked significantly in type 2, but not in type 1 papRCC (**Figures 6B, C**), which is validated also in the testing groups (**Figures 6D–F**). For estimating the predictive power of the genetic characteristics, ROC curves obtained from the papRCC cases were plotted and AUCs of 0.854 and 0.77 were obtained for 3 and 5 years, respectively (**Figure 6G**). The AUC area is getting more pronounced in type 2, but not in type 1 papRCC (**Figures 6H, I**). This has been confirmed in the testing groups as well (**Figures 6J–L**). These data, taken together, indicated that the current gene signature is more suitable for type 2 papRCC. In comparison to papRCC, there was no clear difference between the two groups with respect to survival for the ccRCC cases (**Figure 6M**). The ROC curves obtained for the ccRCC cases were less significant (**Figure 6N**).

### Risk Distribution and Clinically Related Factor in the TCGA Data Set

**Figure 7:**
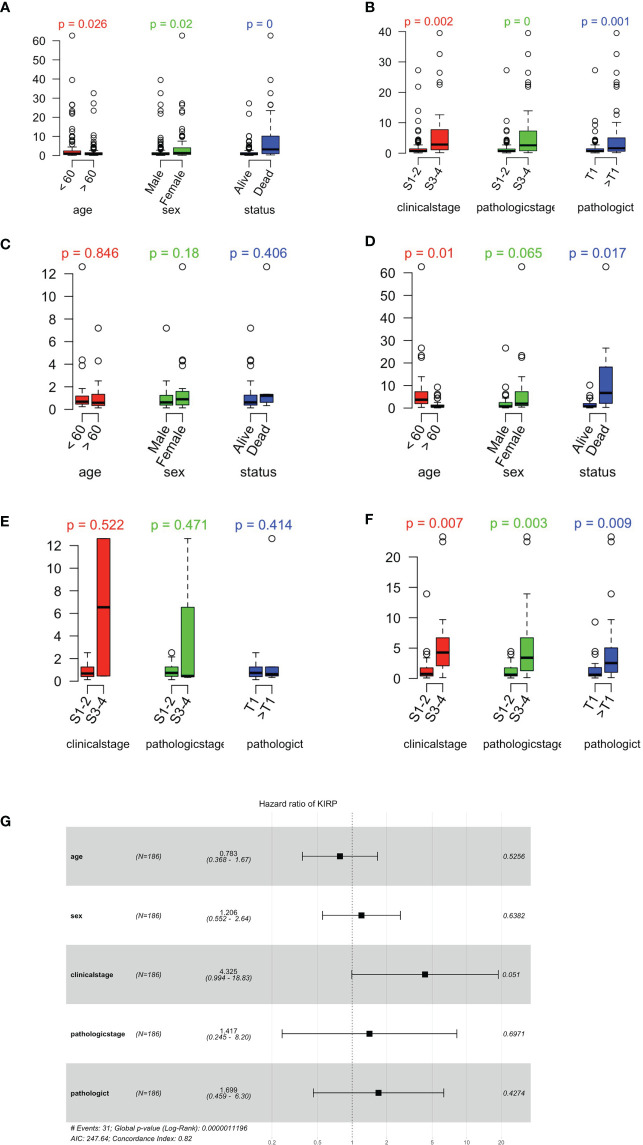
Relationship between risk scores and clinically related characteristics. **(A)** Distribution of risk scores as assessed by age, sex, and survival status in the papRCC. **(B)** Risk score distributions for clinically related stage, pathological stage, and pathological T stage in the papRCC. **(C)** Distribution of risk scores as assessed by age, sex, and survival status in type 1 papRCC. **(D)** Risk score distributions for clinically related stage, pathological stage, and pathological T stage in type 1 papRCC. **(E)** Distribution of risk scores as assessed by age, sex, and survival status in type 2 papRCC. **(F)** Risk score distributions for clinically related stage, pathological stage, and pathological T stage in type 2 papRCC. **(G)** Multiple-variate Cox regression forest plots of risk scores and clinically related characteristics in the GSE2748 data set.

papRCC cases in the TCGA data set were categorized into high- or low-risk groups using the best cutoff values. The box plots shows that not only age, sex and survival status (**Figure 7A**), but also the clinically related stage of the disease and the pathological and pathological T-stages (**Figure 7B**) correlated with the risk scores for individual cases. Furthermore, we also investigated this correlation in the type 1 and type 2 papRCC separately (**Figures 7C–F**). Similar to the survival observation, type 2 papRCC exhibited a tighter correlation with the clinical factors compared with type 1 papRCC. In addition, in order to compare the prognosis to general factors, risk scores for the genetic characteristics and clinically related variables were analyzed by multiple-variate Cox regression investigation. The forest plots showed that age, sex, clinically related stage, pathological stage, and pathological T stage were all correlated with the risk group results (**Figure 7G**). However, the genetic characteristics displayed a higher significance compared with the general risk factors.

### Relationship Between Risk Groups and Immune Checkpoints

**Figure 8:**
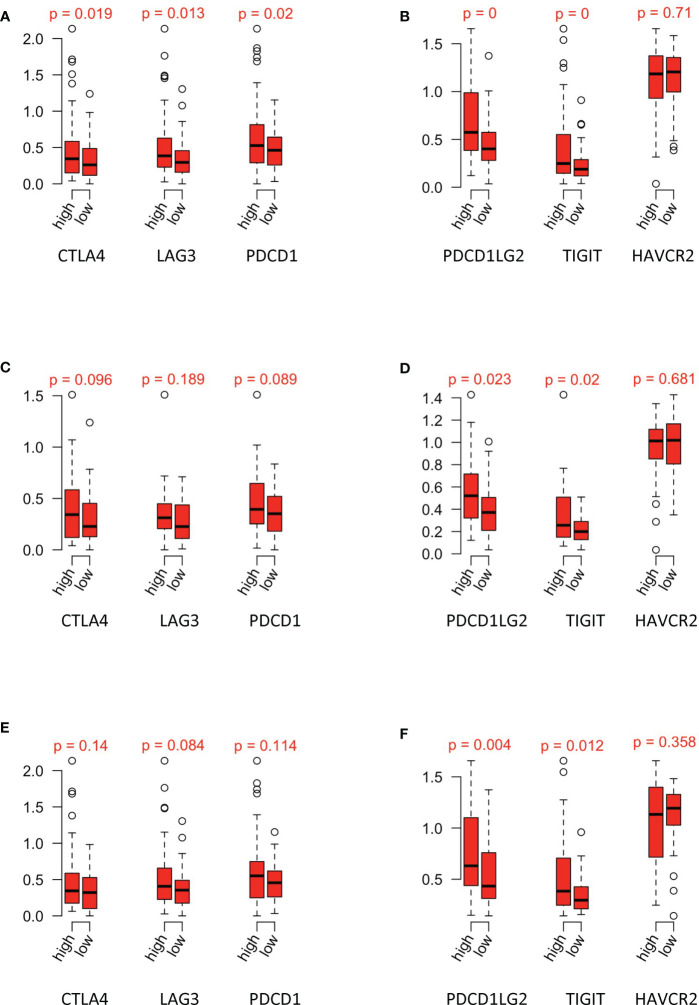
Relationship between risk groups and immune checkpoints. Gene expression of **(A)** CTLA4, LAG3, and PDCD1 and **(B)** PDCD1LG2, TIGIT, and HAVCR2 in the high-/low-risk groups of papRCC. Gene expression of **(C)** CTLA4, LAG3, and PDCD1 and **(D)** PDCD1LG2, TIGIT, and HAVCR2 in the high-/low-risk groups of type 1 papRCC. Gene expression of **(E)** CTLA4, LAG3, and PDCD1 and **(F)** PDCD1LG2, TIGIT, and HAVCR2 in the high-/low-risk groups of type 2 papRCC.

Risk scores of papRCC data and subtypes were evaluated according to gene signatures that were established in the scRNA and TCGA data sets. Correlation of risk scores with immune checkpoints, including CTLA4, LAG3, PDCD1, PDCD1LG2, TIGIT, and HAVCR2, was assessed (**Figures 8A, B**). Type 1 (**Figures 8C, D**) and type 2 (**Figures 8E, F**) data were assessed accordingly. Although all the immune checkpoints showed a tendency of upregulation in the high-risk group, only PDCD1LG2 and TIGIT were significantly increased, in both type 1 and type 2 papRCC, suggesting a correlation of both subtypes with the immune microenvironment.

## Discussion

**Figure 9:**
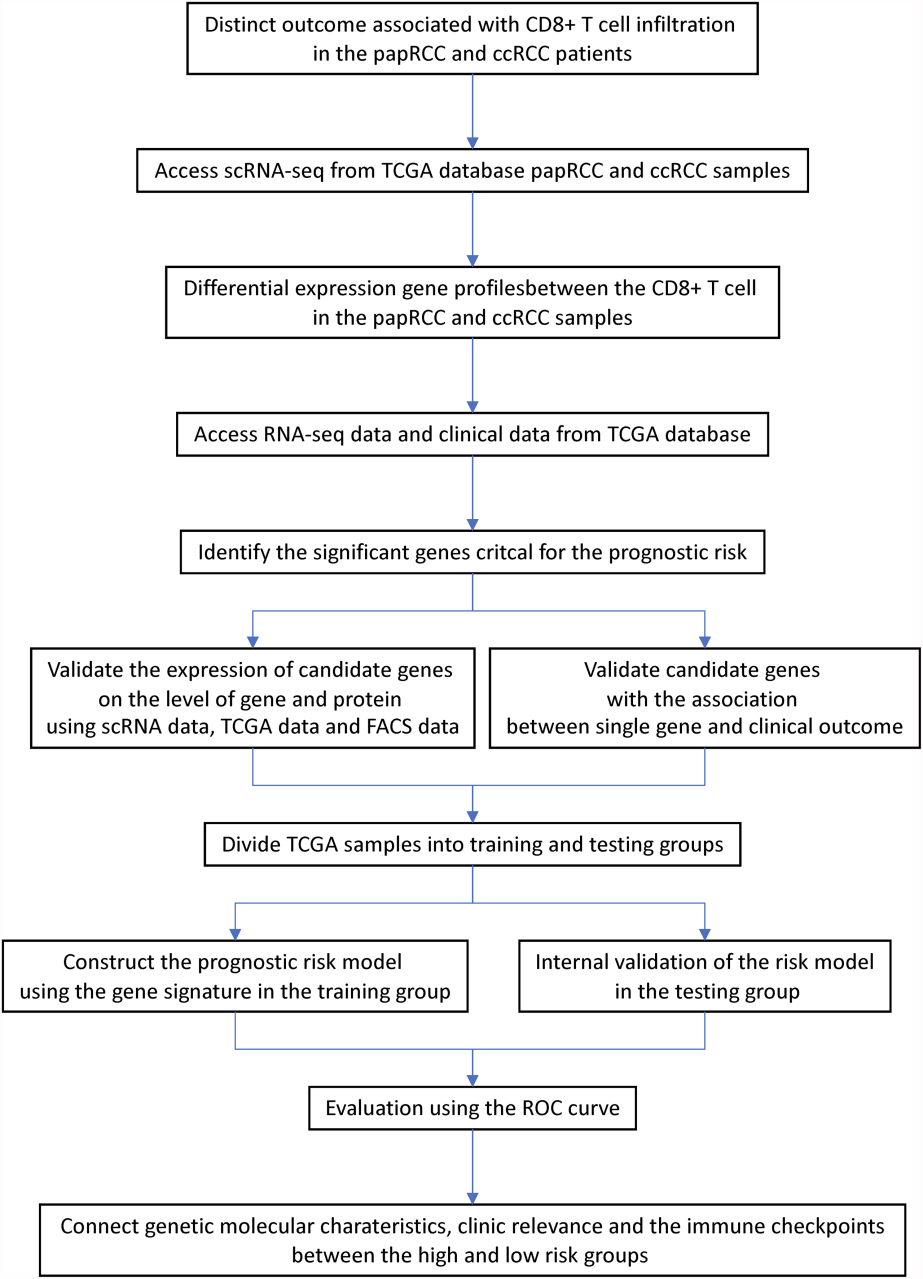
Workflow of the current study.

Using a univariate Cox proportional risk model, we discovered that substantial levels of CD8+ T-cell infiltration were beneficial for cases with ccRCC, whereas they posed a risk for papRCC cases. In this study, we explored the tumor immunization environment using single-cell sequencing and screened for CD8+ T-cell-specific gene features between ccRCC and papRCC. In addition, the researchers built a prognosis genetic signature that divided the overall survival of papRCC into two risk groups. High-risk cases had poorer prognosis. The prognosis gene signature consists of seven genes: four with high risk and three with low risk. Furthermore, the correlation between CD8+ T-cell genetic traits and clinically related parameters was investigated in both TCGA training and testing data sets, to demonstrate the accuracy of genetic traits for prognosis prediction (**Figure 9**). The results showed that risk scores for genetic features were strongly associated with most of the clinically related parameters except for metastasis. In conclusion, multiple-variate Cox regression investigation also indicated that risk scores for genetic characteristics could be used as an independent prognosis factor for papRCC, especially for type 2 papRCC.

The prognosis signature was made up of seven unique genes. Of these genes, the cell growth-regulating protein, LYAR, was often downregulated in tumor-infiltrated lymph nodes (14). YBX-1 levels had been reported in a number of human malignancies and were shown to be associated with poor prognosis and disease recurrence (15). Linc01420 was often upregulated and it could contribute to the progression of pancreatic, nasopharyngeal, and thyroid cancer in cases (16–18). Several groups have previously developed signatures to be used to determine the prognosis of papRCC at the molecular level. However, none of these took into account the tumor immunology of papRCC. Recently, researchers built prognosis signatures of 15 immunization genes to assess survival outcomes of papRCC cases, and the value of immune-correlated prognosis signatures for this disease was shown (19). Another immune-correlated gene signature was built based on immune-correlated gene pairs with a panel of 22 unique genes (4). However, our current prognosis signature is the only one based on CD8+ T cells with scRNA and consists of only seven genes. Therefore, the use of the current gene signature allows the possibility for its combination with the status of CD8+ T-cell infiltration observed in papRCC cases.

It is well known that the successful application of immune checkpoint blockade is attributed to the ability of the antitumor immune response, which largely depends on CD8+ T cells at the site of tumor infiltration. However, renal tumors often exhibit CD8+ T-cell exhaustion (20), which might explain the observation that the high number of CD8+ T cells suggests instead a low overall survival rate of papRCC patients. Bulk RNA sequencing of tumor tissues does not well represent the genomic signature of CD8+ T cells. Therefore, this study explored the tumor immune environment using single-cell sequencing to construct a prognostic genetic signature. Importantly, we found that risk scores were significantly positively correlated with immune checkpoint expression, which may facilitate the screening of patients for immunotherapy.

Our study does have some limitations. More than half of the TCGA samples have unknown subtypes, and therefore, both training and testing data sets in the subtypes consisted of relatively few samples. In addition, our study was retrospective. In the future, researchers need more prospective studies to further apply and validate our findings. Subsequently, researchers need to perform more studies to explore how to simplify the gene signature and how to integrate these findings into existing clinicopathological factors in order to improve the ease of its use and accuracy in clinically related applications.

## Data Availability Statement

The original contributions presented in the study are included in the article/**Supplementary Material**. Further inquiries can be directed to the corresponding authors.

## Ethics Statement

The studies involving human participants were reviewed and approved by Youjiang Medical University for Nationalities, Baise, Guangxi Province, China. The patients/participants provided their written informed consent to participate in this study.

## Author Contributions

JS and LM designed this study. JW, MH, PH, and JZ analyzed the scRNA-seq data. LW, FH, ZW, and SH performed the flow cytometry analysis. YX, JT, GD, and QW analyzed the bulk sequencing data. SS contributed to the statistical analysis and helped compose the manuscript. All authors contributed to the article and approved the submitted version.

## Funding

This research was funded by grants from the National Natural Science Foundation of China (#31970745), Guangxi Natural Science Foundation (#2020GXNSFAA259050 and #2020GXNSFAA259081), Youjiang Medical University for Nationalities (#yy2019bsky001), High-Level Talent Research Projects of the Affiliated Hospital of Youjiang Medical University for Nationalities (#R20196341), and Self-Raised Project of Baise (#20171111).

## Conflict of Interest

The authors declare that the research was conducted in the absence of any commercial or financial relationships that could be construed as a potential conflict of interest.

## Publisher’s Note

All claims expressed in this article are solely those of the authors and do not necessarily represent those of their affiliated organizations, or those of the publisher, the editors and the reviewers. Any product that may be evaluated in this article, or claim that may be made by its manufacturer, is not guaranteed or endorsed by the publisher.

## Supplementary Material

Supplementary Table 1 Supporting data for **Figure 1A**.

Supplementary Data Sheet 1 Supporting data for **Figure 2F**.

Supplementary Data Sheet 2 Supporting data for **Figure 3A**.

Supplementary Data Sheet 3 Supporting data for **Figure 6A**.

Supplementary Data Sheet 4 Supporting data for **Figure 6M**.

Supplementary Data Sheet 5 Supporting data for **Figures 7A, B**.

Supplementary Data Sheet 6 Supporting data for type1 parRCC.

Supplementary Data Sheet 7 Supporting data for type2 parRCC.
